# Terpenic compounds possess anthelmintic and immunomodulatory properties with potential for controlling equine cyathostomin infections

**DOI:** 10.1016/j.ijpddr.2026.100642

**Published:** 2026-04-12

**Authors:** Joshua Malsa, Angélique Chereau, Fabrice Guégnard, Delphine Serreau, Amandine Gesbert, Fabrice Reigner, Léonie Chamoin, Jacques Guillot, Alexandre Vernudachi, Nuria Mach, Géraldine Fleurance, Sonia Lacroix-Lamandé, Andrew R. Williams, Guillaume Sallé

**Affiliations:** a INRAE, Université de Tours, UMR 1282 Infectiologie et Santé Publique, Nouzilly, France; b Department of Veterinary and Animal Sciences, Faculty of Health and Medical Sciences, University of Copenhagen, Dyrlægevej 100, 1870, Frederiksberg C, Denmark; c INRAE, Unité Expérimentale de Physiologie Animale de l'Orfrasière, Nouzilly, France; d Oniris VetAgroBio, 44300, Nantes, France; e INVENesis, UMR 1282 Infectiologie et Santé Publique, Nouzilly, France; f IHAP, Université de Toulouse, INRAE, ENVT, Cedex 3, Toulouse, 31076, France; g Institut français du cheval et de l’équitation, Pôle développement, Innovation et Recherche, Saint-Genès-Champanelle, France; h INRAE, Université Clermont Auvergne, VetAgro Sup, UMR 1213 Herbivores, Saint-Genès-Champanelle, France

**Keywords:** Horse, Strongyle, Nematode, Terpene, Feed additive, Nemabiome

## Abstract

The emergence of anthelmintic-resistant parasite isolates necessitates alternative control strategies. The use of dietary additives with anti-parasitic or immunomodulatory activities has been proposed as a solution, but whether such additives can simultaneously exert dual bioactivities within this context has rarely been explored. Here, we evaluated whether selected terpenes could exert dual bioactivities (anti-parasitic and immunomodulatory) with a view to their use as food additives that can limit strongyle infections in horses. *In vitro* tests with cyathostomin larval development, larval migration, and immune modulation showed that cinnamaldehyde and carvacrol possessed high activity, and nemabiome analysis revealed that both compounds modulated cyathostomin community structure. Moreover, they also displayed significant anti-inflammatory activity in equine mononuclear cells and modulated gene expression induced by parasite antigen in monocytes, including an upregulation of an antioxidant defense network. However, no effects of cinnamaldehyde were observed on parasite egg excretion or host blood cell profiles during a 28 day *in vivo* feeding study. These data suggest that whilst terpenes have broad-acting bioactivities, gaps between *in vitro* properties and *in vivo* efficacy need to be overcome to realize their potential to improve host resistance to parasites.

## Highlights

• Terpenic compounds have anti-parasitic activity against equine strongyles *in vitro*.
• These compounds also modulated parasite community structure *in vitro*.
• Both compounds exerted immunomodulatory effects in equine monocytes.
• Cinnamaldehyde did not lower parasite burdens during a 28 day grazing study.
• Further studies are needed to bridge the gap between *in vitro* and *in vivo*.

## Graphical abstract

**Figure:**
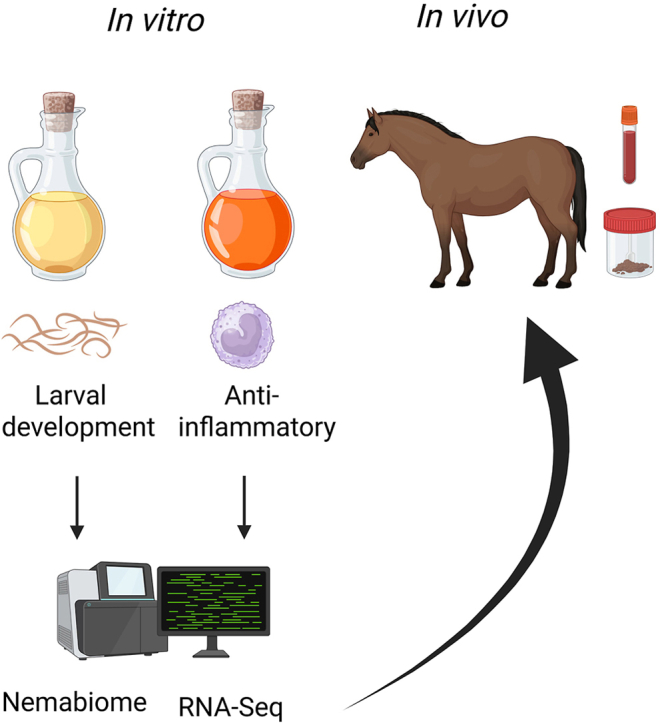

## 1 Introduction

In horses, infection by small strongyles, also called cyathostomins may cause colic, diarrhea, weight loss, and possibly death of the most susceptible hosts, especially young animals (Giles et al., 1985; Love and Duncan, 1992; Love et al., 1999; Peregrine et al., 2014). Nearly all grazing horses are infected by cyathostomins, a complex of 50 described species (Reinemeyer et al., 1984; Lyons et al., 1999; Morariu et al., 2016; Bellaw and Nielsen, 2020) whereby a few keystone species - namely *Cylicocyclus nassatus*, *Cyathostomum catinatum* and *Cylicostephanus longibursatus* - are the most prevalent in the USA (Bellaw and Nielsen, 2020) or in France (Boisseau et al., 2023). A 50 % mortality rate in young horses has been reported in clinical larval cyathostominosis, due to the synchronous reactivation of large numbers of encysted larvae after hypobiosis (Giles et al., 1985; Peregrine et al., 2006; Walshe et al., 2021). Current infection control strategies are based on frequent anthelmintic treatments at regular intervals, but this has resulted in the emergence of drug-resistant populations, which are now widespread (Cai et al., 2024; Abbas et al., 2025; Bull et al., 2025). Due to the lack of novel compounds marketed for horses in the near future, new strategies are turning towards the identification of plants with positive health benefits (Arfuso et al., 2020; Buza et al., 2020; Malsa et al., 2024).

Many plants produce secondary metabolites (SMs) in order to defend against various abiotic and biotic stressors (Bennett and Wallsgrove, 1994; Zaynab et al., 2018; Erb and Kliebenstein, 2020). These molecules are classified into three main groups: alkaloids (or nitrogen compounds), phenolic compounds and terpenes (Bourgaud et al., 2001). Terpenic compounds such as anethole, carvacrol, cinnamaldehyde, eugenol, and thymol have shown *in vitro* activity in inhibiting egg hatching in the small ruminant strongyle *H. contortus* (Zhu et al., 2013; Katiki et al., 2017). *In vivo* activity has also been observed in naturally infected sheep with gastrointestinal nematodes (Ferreira et al., 2016; Štrbac et al., 2022). However, whether these compounds have direct anthelmintic activity against equine parasites or interfere with parasite signaling or development is unclear.

Many plant SMs have also have immunomodulatory activity. For example, the alkaloid compound matrine decreases pro-inflammatory cytokines (TNF-α, IL-6 and prostaglandin E2) in macrophages after stimulation with bacterial lipopolysaccharide (LPS) (Wang et al., 2021). Some phenolic and terpene compounds have shown similar *in vitro* (Srivastava et al., 2009; Tang et al., 2019) or *in vivo* (Wang et al., 2013; Tian et al., 2022) activities. Protective immunity to helminths is dependent on type-2 immune function including production of IgE, eosinophilia, goblet cell hyperplasia, and mucus secretion at the mucosal barrier (Zaiss et al., 2024). These mechanisms have been well established in rodent models, and have been increasingly validated in large animals, including horses where stereotypical components of the type-2 response appear to be associated with infection with cyathostomins or *Strongylus vulgaris* (Dennis et al., 1988; Collobert-Laugier et al., 2002; Steuer et al., 2020). Interestingly, dietary interventions may also modulate immunity to parasites. The ability of plant SMs to suppress oxidative stress and pro-inflammatory, type-1 immune responses may favour the development of type-2 immunity due to the reciprocal counter-regulation of these opposing immune cell subsets. In this way, dietary supplements (e.g. probiotics or cinnamaldehyde) with anti-inflammatory activity may promote anti-helminth immunity through a modulation of cytokine balance and enhanced mucus -production in the intestine (McClemens et al., 2013; Wlodarska et al., 2015; Pinto et al., 2020; Zhu et al., 2022).

Dual action therapeutics are those which simultaneously induce multiple mechanisms of action to exert bioactivity. Such therapeutics may slow the development of drug resistance due to these broad-spectrum bioactivities which target multiple mechanisms in both the parasite and the host. Given the well-described anti-microbial and anti-inflammatory activities of plant SMs, we postulated that diets rich in these compounds may help control parasitic infection by directly targeting nematodes within the gut and/or modulating the host immune response to aid in parasite expulsion. Thus, we aimed to rationally select natural compounds that could serve as ‘dual-action’ feed additives for control of equine cyathostomins. We first screened 6 compounds (*i.e.* anethole, carvacrol, cinnamaldehyde, eugenol, L-menthol, and thymol) for their ability to 1) modulate the inflammatory responses, and 2) to impact larval development and motility. Two promising candidates candidates (carvacrol and cinnamaldehyde) were then used for further studies. Using a metabarcoding approach, their specific effect on cyathostomin species relative abundance and their ability to modulate the activity of equine immune cells was investigated. As there is often a significant gap in the translation of *in vitro* findings to *in vivo* efficacy, we also performed a pilot study to test the *in vivo* effects of dietary cinnamaldehyde supplementation in naturally infected horses. Outcomes included fecal egg counts (FEC), assessment of larval development, and circulating blood immune cell counts.

## 2 Materials and methods

### 2.1 Material and reagents

Fecal, blood, and cyathostomin samples were collected from Welsh ponies from the INRAE experimental farm (doi: 10.15454/1.5573896321728955E12). The procedure received approval from the French Ministry of Research under protocol number APAFIS #35631-202202281021526.

The compounds tested in this study were Anethole, L-menthol, thymol, eugenol, carvacrol or cinnamaldehyde from Pancosma (Z. A. La Pièce 3, 1180 Rolle, Switzerland).

Antigen preparation was produced as described previously (Hellman et al., 2021) 100 mL of cyathostomins third-stage larvae suspension was added to 0.32% sodium hypochlorite solution (Nectra javel 9.6%) for 15 min and 0.48% for 5 min under agitation to exsheath larvae. The larval solution was then centrifuged without brake (5 min, 3000 g) and the supernatant was replaced with sterile H_2_O. This last step was carried out six times to clean the suspension before resuspending it in culture medium (1640 media with 2 mM L-glutamine, 10% FCS and 1% PS) during 30 min (+25 °C). The larval suspension was washed three times as above but in culture medium before being crushed in liquid nitrogen and a small amount of Dulbecco's phosphate buffered saline (DPBS). The larval crushed material was centrifuged without brake (5 min, 3000 g) then filtered (2 μm). The protein concentration in the antigen extract was quantified using a NanoDrop™ (Thermo Fisher Scientific, Waltham, MA, USA). The amount of LPS present in the extract was measured by LCMS^2^ at the Diviomics core facilty (UMS BioSAND 58, Université Bourgogne-Europe, 21000 Dijon France).

### 2.2 Culture, isolation and stimulation of immune cells

#### 2.2.1 Culture and stimulation of murine macrophages

An immunomodulation assay was carried out on RAW 264.7 murine macrophage cells (ATCC TIB-71). Cells were counted and suspended (250,000 cells/mL, 50,000 per well) in 96-well plates (flat bottom) with culture medium composed of RPMI 1640 media with 2 mM L-glutamine (Sigma R5758), 10% inactivated fetal bovine serum (Sigma F2442), 1% PS (100 U/mL penicillin and 100 μg/mL streptomycin (Sigma 4333)). The cells were returned to the incubator (+37 °C, 5% CO_2_) for 2 h, allowing them to adhere to the bottom of the well. After incubation, the cells were pre-stimulated with anethole, L-menthol, thymol, eugenol, carvacrol or cinnamaldehyde (final concentration 5 μg/mL). To evaluate both pro- and anti-inflammatory activities, two experimental setups were used: in the anti-inflammatory condition, the bacterial antigen (lipopolysaccharide – LPS, final concentration 500 ng/mL) was added 30 min after compound exposure, while in the pro-inflammatory condition, an equivalent volume of culture medium was added instead. The cells were then incubated at +37 °C (5% CO_2_) for 24 h. After incubation, the concentration of TNF-α in the medium for each condition was quantified by ELISA using mouse TNF-α paired antibodies (R and D Systems DY410).

#### 2.2.2 In vitro tests on equine PBMCs

To obtain the PBMCs, 20 mL of blood was collected in K2 EDTA tubes (Dutscher 367525A) from four different Welsh ponies. The blood was then diluted 1:1 in sterile DPBS (Sigma D8537) and 20 mL was delicately deposited on 15 mL of histopaque Ficoll-Paque™ PLUS (Sigma 10771). The histopaque blood mix was centrifuged without brake (30 min, 400 g) to isolate the buffy coat located between the serum at the top and the histopaque at the bottom. PBMCs were collected and resuspended in DPBS before red blood cell lysis with lysis buffer (Sigma R7767). Subsequently, PBMCs were treated as RAW 264.7 cells (described above). The TNF-α production was quantified by ELISA using an equine TNF-α Uncoated ELISA Kit (Thermo Fisher Scientific ESS0017).

#### 2.2.3 Isolation and stimulation of equine monocytes

PBMCs were purified as described previously and then incubated with anti-CD14^+^ antibodies coupled to a fluorochrome (Alexa Fluor 647) (Kabithe et al., 2010) for 30 min in the dark (−4 °C). After incubation, monocytes were purified with a cell-sorter (MoFol® Astrios (Beckman-Coulter), IMI-ISP, Nouzilly, France). A minimum of 6 × 10^5^ monocytes per condition was suspended in 96-well plates (flat bottom) in culture media and left to rest (+37 °C, 5% CO_2_) for 17 h. After rest, the cells were stimulated for 6 h under different conditions: by LPS (125 ng/mL), cyathostomins antigen extract (50 μg/mL), dimethyl sulfoxide (DMSO) (0.05%) + LPS (125 ng/mL) and carvacrol or cinnamaldehyde (5 μg/mL) + cyathostomins antigen extract (50 μg/mL).

#### 2.2.4 RNA extraction and transcriptomic analysis of equine monocytes

Following stimulation, cells were collected and subjected to RNA extraction using the Qiagen RNeasy® Plus Mini kit (Qiagen 74134) following manufacturer's recommendations. The quality and purity of RNA was evaluated using bioAnalyzer (Agilent 2100 Bioanalyzer; Agilent Technologies, Santa Clara, CA, USA). RNA was sequenced (200bp paired-end sequencing) using the Illumina NovaSeq 6000 platform (IGATech, Italy). RNAseq data were quality filtered using Trim-galore and the retained reads were pseudo-aligned onto the horse transcriptome (Ensembl110) using the Salmon v. 1.4 software. Count tables were imported using *tximport* function from tximport v.1.28 R package (Soneson et al., 2015) for subsequent modeling and analysis using the DESeq2 v. 1.40.2 R package (Love et al., 2014). Gene ontology (GO) enrichment was conducted using *gost* function from gprofiler2 v. 0.2.2 R package (Raudvere et al., 2019; Kolberg et al., 2020).

### 2.3 In vitro tests on parasites

#### 2.3.1 Larval development assay (LDA)

A larval development assay (LDA) was conducted on a cyathostomins isolate. The eggs were extracted from fecal samples collected from the ground directly after excretion of several ponies and pooled. To avoid contamination, only the top part of the fecal matter (which had not touched the ground) was carefully collected. First, the samples were washed with bottled spring water (Volvic, Danone, Puy-de-Dôme, France) through a colander to remove the largest particles and then through two sieves, a first of 125 μm and a second of 20 μm to retain the eggs. Kaolin (Sigma K7375) was then added to the egg solution before centrifugation. The supernatant was discarded, and the pellet was resuspended in a saturated NaCl solution (density = 1.18) before centrifugation (5 min, 500g RCF). The supernatant was collected on a 20 μm sieve. After egg purification, each well of a 96-well plate was filled with 40 μL of the mixture, resulting in a concentration of 2.75 eggs/μL (110 eggs per well). Thereafter, feces mixture (horse feces mixed with water and pressed through a 125 μm filter, v/v; OD_650nm_ of 0.8), bacteria (*Escherichia coli* OP50 strain) to an OD_650nm_ of 1.3, and 500 μg/mL of amphotericin b (Sigma A4888), were added. The plate was incubated at +25 °C for 24 h, allowing the eggs to develop into L1/L2 larvae. After the incubation, various concentrations of the extracts were added to the wells to establish a dose-response curve, allowing for the determination of the IC50, and incubated for six days. Doses were chosen to approximate the levels of phytonutrients that could be expected in the gut following dietary supplementation – as there is limited information in horses, extrapolation was made from other animal species (Ferreira et al., 2016; Štrbac et al., 2022) Afterwards, undeveloped (L1/L2), or developed (L3) larvae were observed using a 4X objective lens (providing a total magnification of 40X), and the larval development rate was calculated as:

$$\frac{number\;of\;L3}{number\;of\;L1\;and\;L2+number\;of\;L3\;}\times 100$$

The LDA data were used to fit a two-parameter log-logistic regression model to determine inhibitory concentration 50 (IC_50_) (see 2.4 Statistical analysis).

#### 2.3.2 Larval motility assay (LMA)

The INVENesis Migration Trap Assay (MTA) (Harrington et al., 2023) measures the effect of compounds on the third larval (L3) stage of *cyathostomins* isolates*.* Approximately 300 L3s were exposed to the drug or control treatments in each well of a 96 well plate. In all experiments, non ex-sheathed L3s were exposed for 24 h to the compound in a solution of 1.5% of DMSO and 0.00425% of Tween 20. Larvae were then transferred to a migration plate, which is a 96-well plate allowing *cyathostomins* L3s to migrate from a deposit area to a trap area through a corridor. The mobility of the larvae in the trap area was monitored over a 21-min time window using an automated data acquisition system equipped with a Basler acA2000-50gm camera, which measured pixel displacement. The effect of compounds was expressed as a percent reduction of motility compared to negative controls (1.5% DMSO). All compounds were tested in triplicate and the IC_50_ was used to fit a two-parameter log-logistic regression model (see 2.4 Statistical analysis). Following the results of the immunomodulatory activity screen, the LDA and LMA scores were summed to rank the best two extracts.

#### 2.3.3 Measures of differential sensitivity of cyathostomins to carvacrol and cinnamaldehyde

To measure the differential sensitivity of cyathostomins, we performed an LDA test with higher larval content to provide sufficient genetic material. In this case, 5000 larvae (L1/L2) were placed in contact with the extracts in 24-well plates and developed under the same conditions as in section 2.2. After incubation in five different concentrations of carvacrol or cinnamaldehyde, living larvae were collected using a filter (30 μm) and the DNA from 84 samples was individually extracted using NucleoSpin® Tissue kit (Macherey-Nagel, Germany). To determine the better parameter for the analysis, we used a mock community (Malsa et al., 2024). This mock community included 32 nematodes (14 *Cylicocyclus nassatus*, 12 *Cyathostomum pateratum*, 3 *Cylicostephanus goldi*, 1 *Cyathostomum catinatum*, 1 *Cylicostephanus minutus* and 1 *Cylicocyclus ashworthi*). The nemabiome study was performed as described (Malsa et al., 2024). In short, the ITS-2 region was PCR-amplified using NC1 and NC2 primers (Gasser et al., 1993), which included one to three random bases and Illumina adapter overhangs (Courtot et al., 2023). PCR reactions (80 μL) contained HF buffer, dNTPs, primer mix, Phusion High-Fidelity DNA polymerase, and 5 ng/μL genomic DNA. Libraries were prepared from DNA samples (0.4 -74.2 ng/μL in Tris-HCl) and sequenced on two lanes of an Illumina NovaSeq 6000 system (250 cycles; IGATech, Italy). Sequencing reads were trimmed to remove adapters and ITS-2 primers using cutadapt v1.4 (Martin, 2011).

Subsequently, data were analyzed in R v.4.2.1 with the dada2 software (Callahan et al., 2016) using the mock community as a standard to choose appropriate bioinformatic parameters as described previously (Courtot et al., 2023). Using this approach, we retained a maximum number of expected errors of 2 and 5 for the forward and reverse reads respectively, a truncation length of 200 bp, and applied the default BAND_SIZE parameter. Downstream analyses and count tables data were analyzed using the phyloseq v.1.42.0 R package (McMurdie and Holmes, 2013) and vegan v.2.6-4 R package (Oksanen, 2010). All amplicon sequence variants (ASVs) had more than 10,000 hits (min = 216,821) and their occurrence sum higher than 10,000. We utilized a filtration step to remove the samples with an occurrence sum lower than 4000 (no samples removed). Following taxonomy assignment, five false positive ASVs (detected in the mock communities while absent) with a summed relative abundance below 5% across the 142 samples were removed. The final dataset comprised 142 samples and 7 ASVs, corresponding to 17 species.

### 2.4 In vivo evaluation of cinnamaldehyde as a food additive

#### 2.4.1 Animal condition

For the *in vivo* experiment, 13 naturally infected Welsh ponies (between 2 and 5 years old) were used. After period of grazing, the ponies were returned to the stalls two weeks before the experiment to acclimatize to the study conditions. During the acclimatization period, a dietary transition was made from a grass-based diet to one consisting of 600 g of pellets and hay. The ponies were divided into two groups (control (n = 7), cinnamaldehyde (n = 6)) on the basis of age, weight and fecal egg count (FEC) measured one week before the experiment, without significant difference between groups (Table S1).

#### 2.4.2 Experimental design

The trial was conducted over 28 days (from November 8 to December 6 2021). During this period, the ponies in the cinnamaldehyde group receive 50 mg of the additive (23.6 μL of cinnamaldehyde) every day deposited on 20 g of pellets. This was prepared 1 h before consumption. In view of the lack of an emerging tendency, it was decided to double the dose of the additive distributed during the last week (November 29 to Sunday, December 5, 2021). To monitor the antiparasitic and immunostimulatory effects, five fecal samples, blood samples, and weight data were collected per pony over the period of the study (d0, d7, d14, d21, and d28).

#### 2.4.3 Parasitic and immune analyses

Individual FEC data were determined using a modified McMaster technique based on the dilution of 5 g of fecal matter in 70 mL of a saturated NaCl solution (density = 1.18). Eggs were counted using an optical microscope (×150 magnification), the minimum detection limit was set at 50 EPG. The efficacy of cinnamaldehyde was measured with Bayesian hierarchical models (see 2.4 Statistical analysis).

To evaluate the effect of the additive on larval development, 50 g of fecal matter mixed with 15 g of vermiculite (30%) was incubated individually for each horse for 13 days at +23 °C and 70% relative humidity. Infective third-stage larvae (L3) of cyathostomins were then collected using a Baermann apparatus after 24 h and 48 h of sedimentation for each horse and timepoint. The larval count in each sample was determined from 30 drops of 5 μL of the larval solution under the microscope (×40 magnification). The larval development rate was then computed as follows:

$$\left(\frac{Counted\;L3}{FEC\times quantity\;of\;fecal\;matter\;}\right)\times 100$$

Blood samples were analyzed immediately after their collection to determine circulating RBCs and leukocytes by the PFIE INRAE Val de Loire, Nouzilly, France using a Melet Schloesing Laboratoires M-Scan II machine. The complete range of parameters is presented in Supplementary Table S2.

### 2.5 Statistical analysis

All data were analyzed using R software v. 4.3.0. The plant's SM effects on TNF-α levels measured on RAW 264.7 and equine PBMC were statistically analyzed using a Wilcoxon rank sum test with the *wilcox.test* function from the stats v. 4.3.0 package. The larval development and migration rate data from LDA and LMA were used to fit a two-parameter log-logistic regression model to the development and motility ratio, with the percentage of larval development relative to the control as the response variable and the concentration range as the independent variable. We used the *LL.2* function where the lower and upper limits are fixed at 0 and 1 respectively of the drc v. 3.0-1 package. The IC_50_ was determined with *ED* function and statistically compared using the *compParm* function from the same package.

As previously mentioned, all RNAseq data were analyzed using the DESeq2 v. 1.40.2 package and gprofiler2 v. 0.2.2 R package. The abundance and the Shannon diversity dataset were obtained using phyloseq v. 1.44.0 package. The correlation test between Shannon diversity and the extract concentration was realized using the *cor.test* function from the stat v. 4.3.0 package. The Shannon index measured on each group was analyzed using a linear mixed-effects (LME) model with the *lme* function from the nlme v. 3.1-162 package. The treatment group (carvacrol and cinnamaldehyde) was fitted as fixed effect and technical replicate as a random effect. An Generalized Estimating Equations (GEE)model with the *geeglm* function from the geepack v. 1.3.9 package (Højsgaard et al., 2005) was used to statistically analyze the effect of each SM on the cyathostomins count species. The species, the SM concentration and the species x SM concentration interaction were used as fixed effects, and the technical replicate as a random effect.

The data used to determine the groups for the *in vivo* assay (age, weight and FEC at d-7) were analyzed using ANOVA with the *anova* function of stat package. The groups were used as a fixed effect, and horses as a random effect. Individual FEC data were analyzed by a generalized linear mixed-effects models assuming a negative binomial distribution using the *glmer.nb* function of lme4 v.1.1-34 package fitting group (control and cinnamaldehyde), days (d0, d7, d14, d21, and d28), and group x day interaction as a fixed effect, and with horses as a random effect. The larval development rate and hematological parameters (performed individually on each cell population) data were analyzed using LME models. Each dataset was fitted as the *glmer.nb* model used for FEC analysis. The feed additive efficacy was measured using the *fecr_stan* function from eggCount v. 2.3-2 package (Paul et al., 2014) based on the Bayesian hierarchical models recommended by WAAVP guidelines (Kaplan et al., 2023). The average FEC of the treated group at d0 was used as the pre-treatment reference point, and at d7, d14, d21, and d28 as post-treatment, including the correction factor for the McMaster technique. The correlation between hematological data and FEC was determined using the *rcorr* function from Hmisc v. 5.1-0 package.

## 3 Results

### 3.1 Carvacrol and cinnamaldehyde demonstrate potent direct and indirect *in vitro* activity

**Fig. 1:**
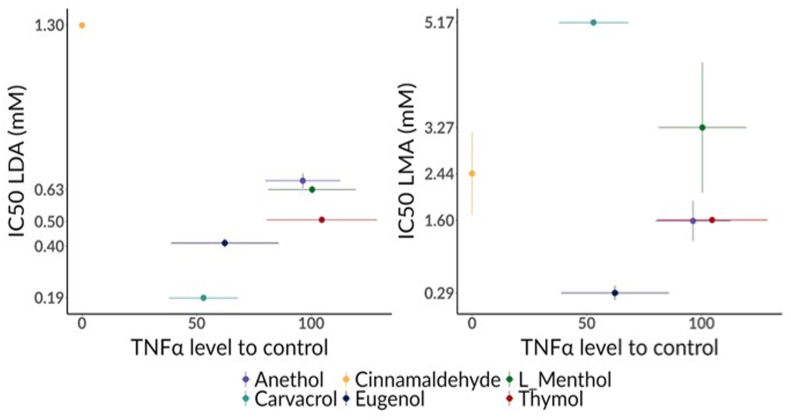
**Scatter plots of the association between TNF-α production and the IC_50_ values (LDA and LMA)** Scatter plots showing the association between TNF-α production percentage (expressed relative to the control) and the IC_50_ values obtained in (left) the Larval Development Assay (LDA) and (right) the Larval Migration Assay (LMA) for six terpene compounds (anethole, cinnamaldehyde, menthol, carvacrol, eugenol and thymol). Each point represents the mean IC_50_ and TNFα production for a given compound, and horizontal/vertical bars indicate the corresponding confidence intervals. Lower IC_50_ values reflect higher antiparasitic potency.

The anti-inflammatory and pro-inflammatory activity of anethole, carvacrol, cinnamaldehyde, eugenol, L-menthol, and thymol was initially screened with the macrophage cell line RAW 264.7 as a robust pre-clinical inflammation model with high reproducibility. The results showed that none of the tested extracts exhibited pro-inflammatory activity, with no TNF-α detected without concurrent LPS stimulation (Fig. S1A). Among the compounds tested, cinnamaldehyde, carvacrol, and eugenol demonstrated the highest anti-inflammatory activity (Fig. 1, and Fig. S1A), significantly reducing TNF-α levels in the culture medium by 100% (*P*_*Bonferroni adjusted*_ < 0.001), 47% (*P*_*Bonferroni adjusted*_ = 0.002) and 38% (*P*_*Bonferroni adjusted*_ = 0.04), respectively, compared to the LPS-stimulated control. To assess whether these compounds also exerted direct antiparasitic activity against equine strongyles, LDA and LMA were then performed to identify the most active extracts using the IC_50_, defined as the concentration required to reduce parasite development or migration by 50% (Fig. S1B and C). The two extracts with the best activity in inhibiting larval development were carvacrol (IC_50_ = 0.19 mM [95% c.i. = 0.17-0.20]) and eugenol (IC_50_ = 0.41 mM [95% c.i. = 0.38-0.45]) (Fig. 1). In terms of motility inhibition activity, eugenol (IC_50_ = 0.29 mM [95% c.i. = 0.04-0.54]) and anethole (IC_50_ = 1.59 mM [95% c.i. = 0.87-2.31]) were the two most effective. Following integration of both assays into a composite activity score, cinnamaldehyde and carvacrol obtained the best rankings, with scores of 3.74 and 58.35, respectively. All results are summarized in Supplementary Table S3.

**Fig. 2:**
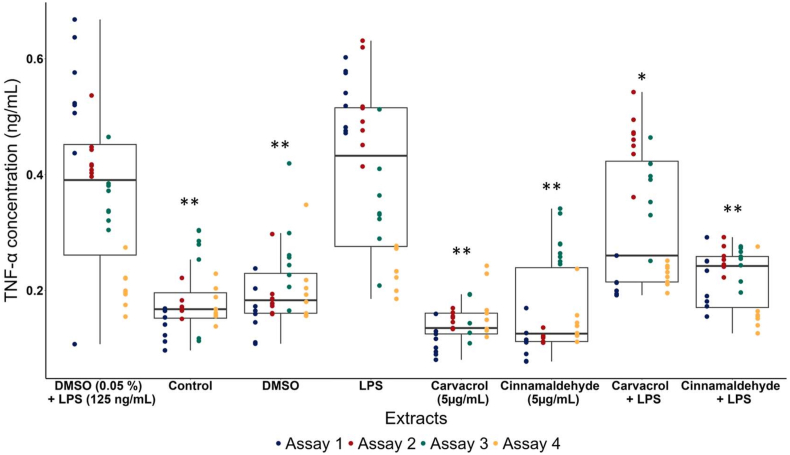
**Anti-inflammatory activity of carvacrol and cinnamaldehyde on equine PBMC** Boxplots showing TNF-α concentrations (ng/mL) measured in equine peripheral blood mononuclear cells (PBMCs) exposed to DMSO (0.05%), LPS (125 ng/mL), the combination of DMSO and LPS, carvacrol (5 μg/mL), cinnamaldehyde (5 μg/mL), the combination of either compound with LPS, and the untreated condition (control). Points represent individual replicates from four independent assays. Asterisks indicate significant differences relative to the corresponding control condition (∗*P* = 0.01, ∗∗*P* < 0.001).

Following the results of the screening on RAW 264.7 cells, the anti-inflammatory activity of cinnamaldehyde and carvacrol was evaluated on equine PBMCs to ensure cross-species translatability. Across four independent trials with seven technical replicates each, cinnamaldehyde and carvacrol reduced TNF-α levels by 41% (*P* < 0.001) and 14% (*P* = 0.01), respectively (Fig. 2). Thus, these results indicate that both compounds can modulate equine immune function as well as inducing direct anti-parasitic effects against equine nematodes.

### 3.2 Larval community structure variation following exposure to carvacrol, cinnamaldehyde and pyrantel

We further analyzed changes in larval community structure following exposure to concentration gradients of cinnamaldehyde, carvacrol, or pyrantel (used as a reference anthelmintic) to identify putative species-specific effects. The tested concentrations of cinnamaldehyde and carvacrol were based on those used in the previous *in vitro* LDA test.

Under control condition (no compound exposure), 15 species were correctly identified, two were identified at the genus level, and one could only be identified at the family level, with an average count of 407 individuals per sample. Four species were dominant, each displaying an absolute abundance mean higher than 5000 individuals: *Cyathostomum catinatum* (5,444), *Cyathostomum pateratum* (51,090), *Cylicocyclus ashworthi* (84,809), and *Cylicocyclus nassatus* (54,748) (Fig. S2).

**Fig. 3:**
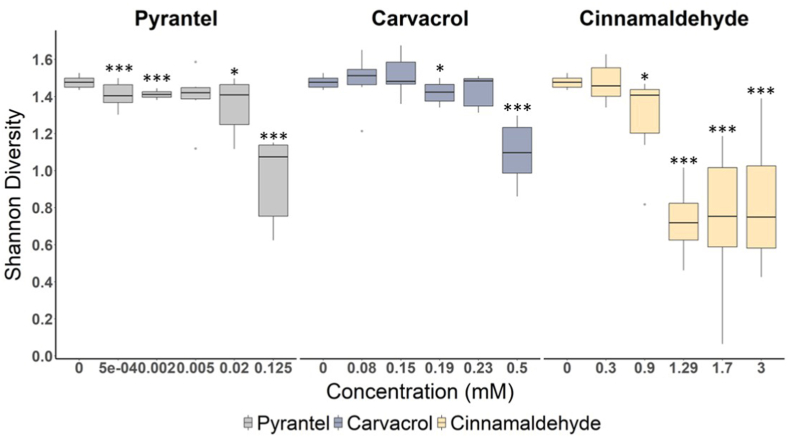
**Shannon diversity of larval community following exposure to pyrantel, carvacrol, and cinnamaldehyde** Boxplots showing Shannon diversity measured in Cyathostomin larval community exposed to an increasing gradient of pyrantel (grey), carvacrol (blue) and cinnamaldehyde (yellow). The asterisk indicates the significant difference (*P* < 0.01) of Shannon diversity at the specific concentration for each condition (carvacrol or cinnamaldehyde).

**Fig. 4:**
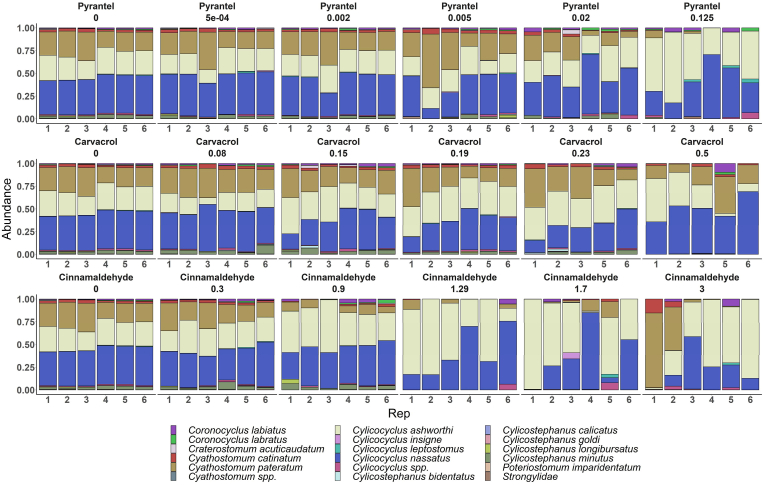
**Cyathostomin larval community abundance estimated using the metabarcoding approach during exposure to pyrantel, carvacrol and cinnamaldehyde.** Relative abundance of Cyathostomin larval species across increasing concentrations of pyrantel (top row), carvacrol (middle row), and cinnamaldehyde (bottom row). Each compound was tested at six concentrations, with six replicates per condition. Bar plots represent species-level community composition, with colors indicating individual species (see legend).

Exposure to increased concentrations of cinnamaldehyde, carvacrol, or pyrantel resulted in a significant decrease in alpha diversity, as measured by the Shannon index. This decline was strongly correlated with the increase of cinnamaldehyde (r_Pearson's_ = −0.64; *P* < 0.001), carvacrol (r_Pearson's_ = −0.68; *P* < 0.001), and pyrantel (r_Pearson's_ = −0.84; *P* < 0.001) concentration. Compared with the control group, the Shannon index was significantly lower at the highest concentration of carvacrol and pyrantel (*P* < 0.001) (Fig. 3), and for the three last cinnamaldehyde concentrations evaluated (*P* < 0.001). This dose-dependency implies a specific response of the parasite community to drug pressure. Moreover, species-specific responses were also observed. Under cinnamaldehyde exposure, only *C. ashworthi* showed a significant increase in abundance at the highest concentration tested (3 mM*, P* < 0.001) (Fig. 4). In the face of the carvacrol concentration gradient, the counts of *C. nassatus* became significantly higher at the last concentration tested (0.5 mM) compared with the control, from 80,229 to 105,289 (*P* = 0.03) (Fig. 4). In contrast, pyrantel exposure led to a significant reduction of *C. pateratum* at 0.125 mM (from 40,005 to 4075, *P* < 0.001). Collectively, these data demonstrate that phytochemicals not only exert anti-parasitic activity but also modulate the species composition of the parasite communities. Notably, the patterns of community modulation induced by cinnamaldehyde and carvacrol differed from those observed with the anthelmintic drug pyrantel, indicating that these plant compounds may exert distinct, species-specific effects.

### 3.3 Cinnamaldehyde and carvacrol activate genes regulating oxidative stress in equine monocytes exposed to larval antigen

**Fig. 5:**
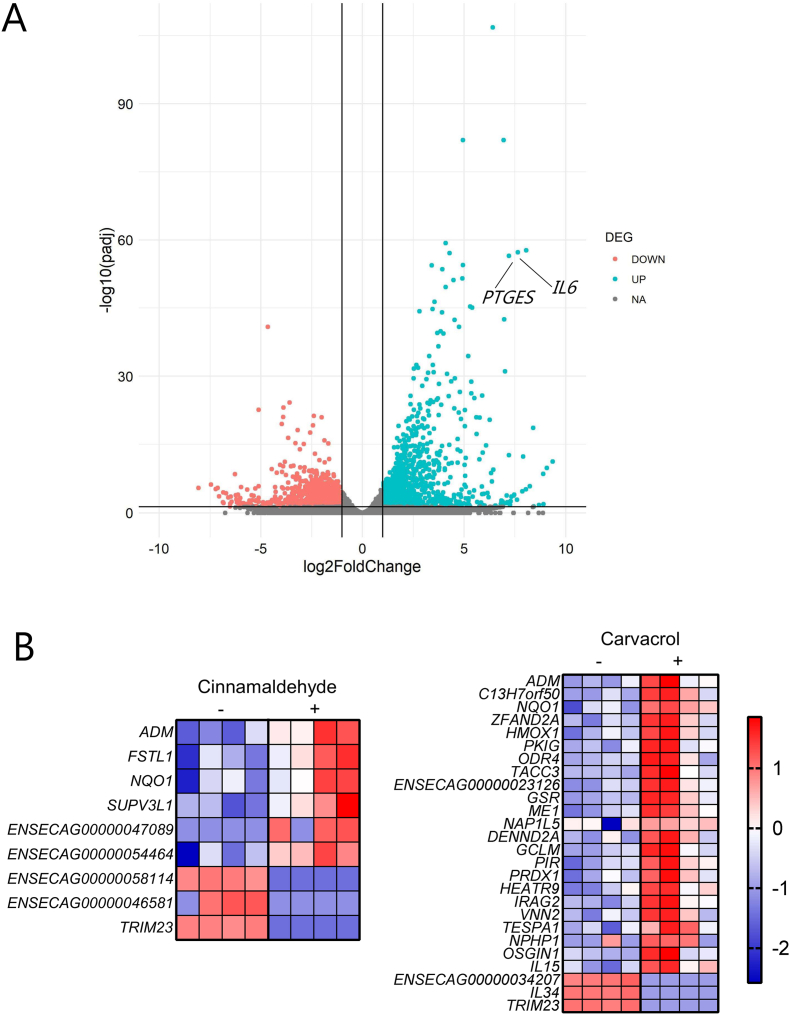
**Gene expression changes induced by parasite antigen in equine monocytes** A) Volcano plot showing genes up- or down-regulated (adjusted *p* value < 0.05) in equine monocytes exposed to cyathostomin antigen B) Expression levels (Z-scores based on normalized read counts) of genes differentially regulated (adjusted *p* value < 0.05) in monocytes treated with antigen combined with cinnamaldehyde or carvacrol, relative to antigen alone. The heat map indicates the expression level with red indicating a high Z score and blue a lower Z score.

Given the apparent immunomodulatory properties of the plant-derived compounds, we next investigated whether they could influence the response of equine immune cells to parasite antigens. To this end, monocytes were isolated from horse blood and were stimulated with different treatments. We first confirmed that the monocytes responded robustly to LPS, and that different solvents (water or DMSO) had negligible effects on gene transcription (Fig. S3). LPS stimulation triggered a significant change in the expression of 666 genes. Among these, 526 genes were upregulated, showing significant enrichment in immune-related pathways such as the cytokine-cytokine receptor interaction (KEGG:04060, *P adjusted* = 1.4e-9), TNF response (KEGG:04668, *P adjusted* = 1.0e-08), Nf-Kappa B (KEGG:04064, *P adjusted* = 2.2e-7), JAK-STAT signaling (KEGG:04630, *P adjusted* = 4.4e-07) or Toll-like receptor (TLR) signaling pathway (KEGG:04620, *P adjusted* = 0.1e-03). Thus, the isolated monocytes responded with a classical pro-inflammatory response to the TLR4 agonist LPS. Stimulation with parasite antigen alone elicited a similar transcriptional response, with more than 1000 genes differentially expressed compared to the solvent only control. Notably, these included inflammatory genes such as *IL6* and *PTGES* encoding prostaglandin synthase, suggesting that the parasite antigens drove an inflammatory-type response in equine monocytes (Fig. 5A). However, we noted that there was also measurable LPS in the parasite antigen preparation (>100 ng/mL), which may also contribute to the inflammatory response. To see if this response was modulated by cinnamaldehyde or carvacrol, we compared gene expression in cells stimulated only with parasite antigen, or with concurrent treatment with either of the two phytochemicals. Relative to antigen alone, cinnamaldehyde treatment regulated the expression of 9 genes and carvacrol 26 genes (adjusted *p* value < 0.05). Notably, both compounds strongly suppressed the expression of *TRIM23*, involved in inflammatory responses to pathogen infection, whilst upregulating numerous genes involved in antioxidant defenses including *NQO1*, and in the case of carvacrol *HMOX1*, *PRXD1* and *GCLM* encoding molecules involved in peroxidase sand glutathione metabolism (Fig. 5B). Thus, both cinnamaldehyde and carvacrol could modify the response of horse immune cells to antigenic challenge, with a dominant response upregulation of an antioxidant network that may limit inflammation and promote protective immunity.

### 3.4 In vivo trial of cinnamaldehyde as a feed additive

**Fig. 6:**
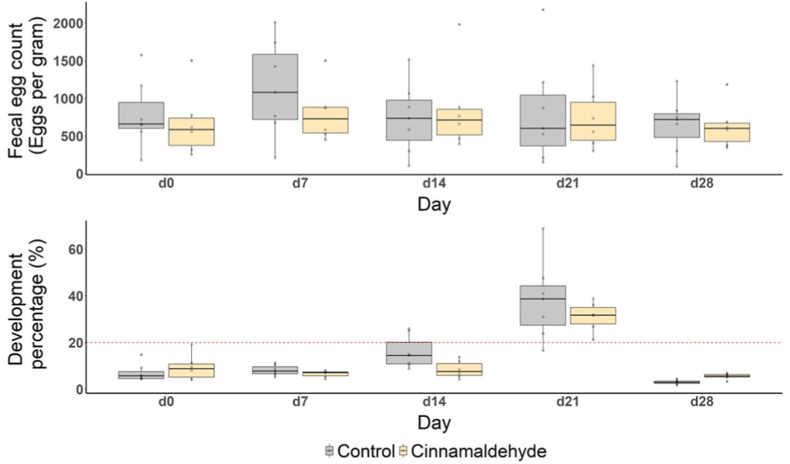
**Fecal egg count (top panel) and larval development rate (bottom panel) for ponies eating cinnamaldehyde (yellow)-based additives compared to the control group (grey)** Impact of cinnamaldehyde treatment on fecal egg count (top panel) and larval development (bottom panel) over a 28-day period. Box plots compare control (grey) and cinnamaldehyde-treated (yellow) groups at five time points (days 0, 7, 14, 21, and 28). The red line in the bottom panel indicates the 20% development rate.

**Fig. 7:**
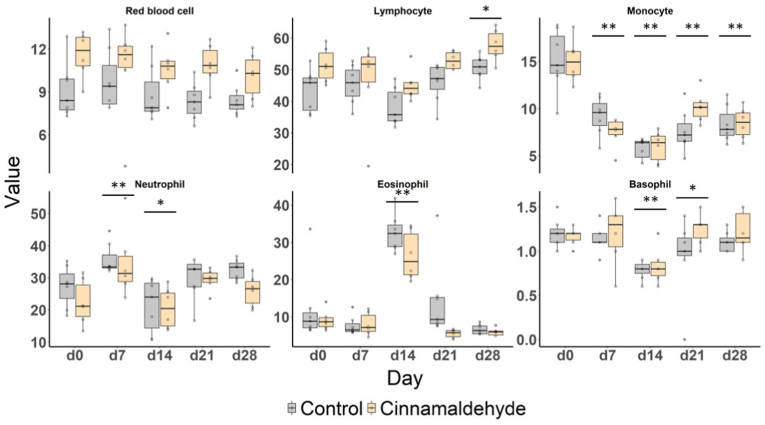
**Trends in six hematological parameters selected during the *jn vivo* intervention study** Temporal effects of cinnamaldehyde treatment on hematological parameters over a 28-day period. Box plots compare control (grey) and cinnamaldehyde-treated (yellow) groups across five time points (days 0, 7, 14, 21, and 28) for six blood cell types: red blood cells, lymphocytes, neutrophils, eosinophils, monocytes, and basophils. The horizontal bars with the stars indicate a significant difference of ∗ *P* ≤ 0.05 and ∗∗*P* ≤ 0.01 in the days compared to the other group, specific to the cell population.

Based on consistently high bioactivity in anti-parasitic and anti-inflammatory tests, cinnamaldehyde was chosen for a controlled feeding intervention in horses. Thus, one group of horses received oral cinnamaldehyde supplementation, while a control group did not, in order to *in vivo* investigate the effects previously observed *in vitro*. The administration of cinnamaldehyde as a feed additive had no effect on parasite parameters throughout the *in vivo* trial (Fig. 6). A transient difference in FEC was observed on day 7, with significantly higher values in the control group compared to the cinnamaldehyde group (*P* = 0.05), likely due to the increased excretion in the control group rather than a treatment effect. Overall, there was no efficacy of cinnamaldehyde with a maximum reduction of only 4.9% at d28 (Table S4). Similarly, no consistent effects were observed on the selected hematological parameters (Fig. 7). However, some cell populations showed significant differences between the average cell count per day. This was the case for the number of lymphocytes at d28 (*P* = 0.01), neutrophils at d7 (*P* = 0.006) and eosinophils at d14 (*P* < 0.001), where the average count was higher than at d0. However, the average number of basophils at d14 and d21, as well as neutrophils at d14, was significantly lower than at d0 (*P* = 0.0007, 0.03, 0.05, respectively). No significant correlations were found between FEC and cell count (Table S5). No differences in body weight were observed in the study.

## 4 Discussion

This study provides a multifaceted evaluation of the antiparasitic and immunomodulatory potential of selected plant-derived terpenes -specifically cinnamaldehyde and carvacrol, -against equine cyathostomins. Through a combination of *in vitro* parasitological assays, immune cell profiling, transcriptomic analysis, and a preliminary *in vivo* trial, we explored both the direct and indirect mechanisms by which these compounds may contribute to parasite control.

To our knowledge, our study is the first to evaluate the sensitivity of cyathostomins to these terpenic molecules. Previous work on *C. elegans* found IC_50_ values of 1.45 nM for eugenol and 0.80 nM for carvacrol, respectively (Hernando et al., 2019). Compared to our results, cyathostomins are more sensitive to eugenol and carvacrol, with IC_50_ values of 0.29 mM and 0.19 mM, respectively. Conversely, cinnamaldehyde showed lower potency against cyathostomin motility, with an IC_50_ of 1.3 mM (0.16 mg/mL) compared to its reported effects on *Haemonchus contortus* (IC_50_ = 0.018 mg/mL (Katiki et al., 2017) or IC_50_ = 7.2 μM(Frota et al., 2023)) and *Ascaris suum* (Williams et al., 2015) where 125 μg/mL cinnamon bark extract completely inhibited larval migration. Combined with our results showing a modification of cyathostomin population structure, our findings suggest that whilst these phytochemicals exert selective pressure on cyathostomin communities, certain species may be more resilient or better adapted to the molecules tested. Further studies will be necessary to determine in detail these differential susceptibilities, and also to evaluate the possibility that these intrinsic differences may confer a selection advantage to more tolerant species that may become dominant in the nemabiome over time.

The second aspect of this study was to investigate the indirect immunomodulatory activity of the selected terpenic compounds. A first evaluation of the anti-inflammatory activity of cinnamaldehyde and carvacrol on equine peripheral blood mononuclear cells was performed. While both compounds demonstrated anti-inflammatory activity on equine cells, the magnitude of the response was lower than that observed in murine macrophage models, likely due to the inherently lower reactivity of the equine primary cells. To further investigate the effects of these two compounds on the horse's immune system, we described, for the first time, the transcriptional response of equine monocytes stimulated with cyathostomin larval antigens, in the presence or absence of cinnamaldehyde or carvacrol. This builds on previous work (Hellman et al., 2021) who evaluated cytokine expression by equine PBMC, after *Strongylus vulgaris* or cyathostomin antigen stimulation. L3 cyathostomin antigen induced over-expression of IL-4 and IL-13. In our study, stimulation with L3 cyathostomin antigens induced differential expression of 750 genes in equine monocytes, of which 468 up and 282 down-expressed. Pathway enrichment analysis using the KEGG database, revealed significant activation of immune-related pathways, including TNF and TLR signaling pathway, both of which are central to innate immune activation and cytokine production.

In our transcriptomic analysis, LPS stimulation induced differential expression of 666 genes compared with the control, including 140 genes under-expressed and 526 over-expressed. Functional enrichment analysis revealed strong activation of immune-related pathways including cytokine-cytokine receptor interaction, TNF, NF-κB, and JAK-STAT signaling pathway. This is consistent with known mechanisms in human and murine models, where TLR4 recognizes LPS and activates NF-ϰB, leading to monocyte differentiation into pro-inflammatory macrophages (Guha and Mackman, 2001; Wan et al., 2016). The TLR signaling pathway was also found to be overexpressed in parasite antigen-stimulated monocytes. These data suggest that cyathostomin larval antigens similarly trigger monocyte activation and polarization toward a M1 phenotype, as evidenced by the upregulation of TNF, NF-κB and HIF-1A pathways. More, the NF-κB pathway has been described as able to polarize M2-type macrophages, specific for repair following infection (Notari et al., 2014; Cosín-Roger et al., 2016), into M1 macrophages (Hagemann et al., 2009). This supports the hypothesis that parasite antigens can elicit a robust inflammatory response in equine innate immune cells. However, interpretation of these results is complicated by the fact that we observed LPS contamination in our parasite antigen preparation, so we cannot rule out the pro-inflammatory responses derive from this contamination. Regardless, it was interesting to note that the terpene compounds appeared to suppress this inflammatory response, providing for the first time evidence that plant SMs have anti-inflammatory activity in horse immune cells. Whilst the gene changes were modest, they were closely linked to anti-inflammatory and antioxidant mechanisms that have been well described for these compounds in other animal species, suggesting a conserved function of these molecules in modulating oxidative stress and inflammation (Alfawaz et al., 2025; Yu et al., 2025). The intrinsic anti-inflammatory responses of the terpenes has clear relevance for animal health is chronic inflammation is a significant issue in livestock including horses, however whether this response either contributes to or limits the development of protective immunity to helminth infection is not clear. However, it is interesting to note that pro-inflammatory responses in monocytes and macrophages are often associated with suppression of type-2 immunity, and thus the ability of the phytochemicals to limit this inflammatory response and instead promote an antioxidant regulatory network may suggest the potential to promote the development of parasite-specific immunity. Further studies should focus on exploring the connection between modulation of inflammation and promoting resistance to parasites. Whilst this phenomenon is increasingly well described in rodent models (Alhallaf et al., 2018; Oliveira et al., 2022), each host-parasite system needs to be evaluated empirically and further clinical trials in horses will be necessary.

Following the promising *in vitro* activity screening results showing anti-parasitic and immunomodulatory properties, an *in vivo* study was conducted over a 28-day period to evaluate the effects of cinnamaldehyde administered as a feed additive, on parasitological and hematological parameters in horses. To date, this is the first study evaluating the *in vivo* antiparasitic potential of this compound in horses. We must acknowledge that this was a pilot study with a small number of horses, and that larger cohort trials will be necessary to derive a firm conclusion on the *in vivo* efficacy of these molecules. Despite this, it does appear from our preliminary data that there is a significant *in vitro* to *in vivo* translation gap that needs to be overcome. Despite increasing the dosage during the final week of the study, no significant antiparasitic effects directly associated with cinnamaldehyde were observed. Although the observed number of eggs excreted at d7 was significantly lower in the test batch compared with the control, this result was likely due to a transient increase in FEC excretion in the control group rather than a treatment effect. This was also confirmed by the efficacy of the additive measured negatively at this date and did not exceed 4.9% at d28. These findings highlight the well-documented discrepancy between *in vitro* and *in vivo* results. In our case, once ingested by the animal, the food additive has to pass through the digestive tract before coming into contact with the parasite in the hindgut. During this passage, several physiological factors can influence the structure, stability, and bioavailability of active molecules, including digestive pH, gastric emptying speed, and secretion, as well as interactions with dietary components (Vertzoni et al., 2019). In addition, the gut microbiota play a crucial role in modulating the fate of these compounds. Microbial enzymes can metabolize phytochemicals into bioactive or inactive forms, thereby altering their pharmacokinetics and physiological effects. This microbial transformation adds another layer of complexity to the *in vivo* efficacy of plant-derived additives and highlights the importance of considering host–microbiota interactions in the development of nutraceutical strategies. Moreover, whilst in this pilot study we based the inclusion rate on existing commercial phyto-therapeutic doses for horses, studies to determine the pharmacokinetic properties of these compounds in equids would be highly relevant to inform future efforts to explore the *in vitro* and *in vivo* anti-parasitic activity.

The transition from *in vitro* to *in vivo* therefore requires galenic studies to improve molecule delivery. In the context of equine parasitology, the development of an *in vitro* equine fermenter capable of reproducing hindgut conditions (Strauch et al., 2017) could help identify the fate of ingested molecules. Some authors have also proposed the use of yeast particles offering stability and prolonged release properties for essential oil use (Soto et al., 2023).

In summary, while cinnamaldehyde and carvacrol demonstrated promising *in vitro* antiparasitic and immunomodulatory activity, the *in vivo* activity of cinnamaldehyde was limited under the tested conditions. Future studies should focus on optimizing formulation and delivery strategies to improve compound stability and bioavailability in the equine gastrointestinal tract. This will be essential to fully harness the therapeutic potential of plant-derived compounds in parasite control programs.

## CRediT authorship contribution statement

**Joshua Malsa:** Writing – review & editing, Writing – original draft, Methodology, Investigation, Formal analysis, Conceptualization. **Angélique Chereau:** Writing – review & editing, Investigation. **Fabrice Guégnard:** Writing – review & editing, Investigation. **Delphine Serreau:** Writing – review & editing, Investigation. **Amandine Gesbert:** Writing – review & editing, Investigation. **Fabrice Reigner:** Writing – review & editing, Investigation. **Léonie Chamoin:** Writing – review & editing, Investigation. **Jacques Guillot:** Writing – review & editing, Supervision. **Alexandre Vernudachi:** Writing – review & editing, Investigation. **Nuria Mach:** Writing – review & editing, Supervision, Methodology, Conceptualization. **Géraldine Fleurance:** Writing – review & editing, Supervision, Methodology, Conceptualization. **Sonia Lacroix-Lamandé:** Writing – review & editing, Supervision, Methodology. **Andrew R. Williams:** Writing – review & editing, Supervision, Methodology, Conceptualization. **Guillaume Sallé:** Writing – review & editing, Supervision, Methodology, Conceptualization.

## Conflict of interest statement

The authors have no financial or other conflicts of interest to declare.

## Data Availability

ITS2 sequence data is available at https://doi.org/10.57745/HKJLJG. RNA-Seq data is available at GEO under accession number GSE312280.
