# Supplementary material for: Terpenic compounds possess anthelmintic and immunomodulatory properties with potential for controlling equine cyathostomin infections
Source: Int J Parasitol Drugs Drug Resist. 2026 Apr 12;31:100642. doi: 10.1016/j.ijpddr.2026.100642 (PMC13101707; doi:10.1016/j.ijpddr.2026.100642)
Supplement: Multimedia component 1 [file mmc1.pdf]

Supplementary Information for:

Terpenic compounds possess anthelmintic and immunomodulatory properties with potential for controlling equine cyathostomin infections

Contents:

Supplementary Tables 1-5

Supplementary Figures 1-4

Supplementary Table S1. Batches characteristics

	Control	Cinnamaldehyde
Age (mean ± s.d)[1]	3.7 ± 1.60	3.0 ± 1.09
Weight (mean ± s.d)	285 ± 46	286 ± 55
FEC (mean ± s.d)	626 ± 268	615 ± 387

[1] Mean and standard deviation of age, weight and FEC per group

Supplementary Table S2. Summary of the *in vivo* screening data

Parameters	Unit
White blood cells	m/mm3
Lymphocytes	%
Monocytes	%
Neutrophils	%
Eosinophils	%
Basophils	%
Others (cellular debris)	%
Red blood cells	m/mm3
Mean corpuscular volume (MCV)	fl
Hematocrit	%
Mean corpuscular hemoglobin (MCH)	pg
Mean corpuscular hemoglobin concentration (MCHC)	g/dl
Hemoglobin	g/dl
Red cell distribution width - absolute (RDW)	fl
Red cell distribution width - relative (RDW-CV)	%
Microcytes	%
Macrocytes	%
Platelets	m/mm3
Mean platelet volume (MPV)	fl
Plateletcrit (PCT)	%
Mode	fl
Median	fl
Platelet distribution width (PDW)	-
Microplatelet	%
Macroplatelet	%

Supplementary Table S3. Summary of the *in vitro* screening data

Extract	TNFα level to the control (%)	LDA IC50 (mM) [Lower ; Upper]	LMA IC50 (mM) [Lower ; Upper]	Score[1]
Anethole	96.3	0.66 [0.61 ; 0.72]	1.59 [0.87 ; 2.31]	98.55
Carvacrol	52.99	0.19 [0.17 ; 0.21]	5.17 [n.d[2] ; n.d]	58.35
Cinnamaldehyde	0	1.3 [1.27 ; 1.32]	2.44 [0.98 ; 3.91]	3.74
Eugenol	62.3	0.41 [0.38 ; 0.45]	0.29 [0.04 ; 0.54]	63
L Menthol	100.37	0.63 [0.60 ; 0.66]	3.27 [0.96 ; 5.59]	104.27
Thymol	104.61	0.51 [0.49 ; 0.52]	1.61 [n.d ; n.d]	106.73

[1]The score is the sum of each column (TNFα level to the control + LDA IC_{50} + LMA IC_{50})

[2]Not determined

Supplementary Table S4. Cinnamaldehyde efficacy during the trial

Day	Cinnamaldehyde
d7	-20.9[1] [-36.4 ; -4.6][2]
d14	-29.4 [-47.4 ; -12.7]
d21	-12.1 [-27.3 ; 2.6]
d28	4.9 [-9.2 ; 17.8]

[1]Efficacy, [2]90% credibility interval

Supplementary Table S5. Correlation data

Correlation matrix

P values		FEC	Red blood cell	Lymphocyte	Monocyte	Neutrophil	Eosinophil	Basophil
	FEC		-0.06	-0.03	0.03	0	0	-0.01
	R.B.C.	0.5331		0.21	0.1	-0.09	-0.11	0.32
	Lympho	0.8098	0.0439		0.11	-0.32	-0.58	0.32
	Mono.	0.7849	0.3448	0.289		-0.07	-0.41	0.49
	Neutro.	0.9962	0.3794	0.0015	0.5097		-0.5	0.19
	Eosino	0.965	0.2993	0	0	0		-0.59
	Baso	0.9251	0.0014	0.0013	0	0.0587	0	

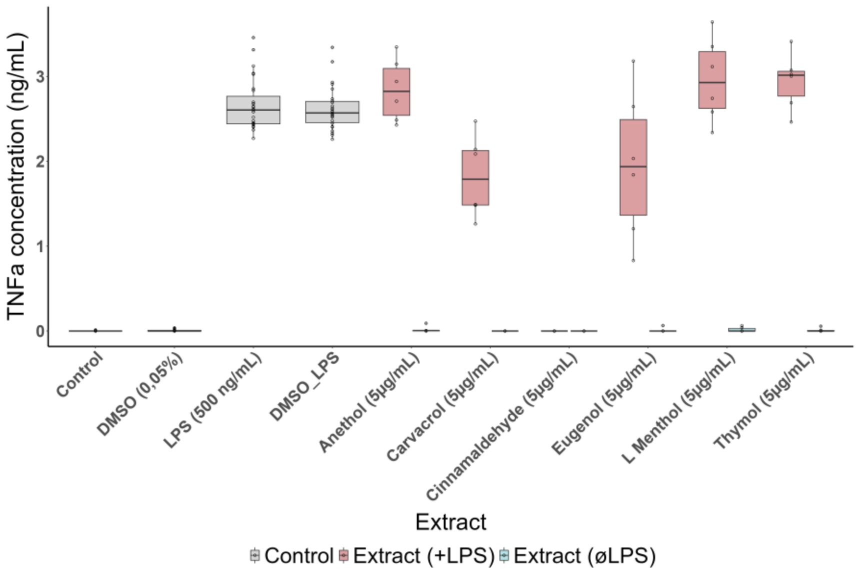
TNFa concentration (ng/mL)
0
1
2
3
Control
DMSO (0,05%)
LPS (500 ng/mL)
DMSO_LPS
Anethol (5µg/mL)
Carvacrol (5µg/mL)
Cinnamaldehyde (5µg/mL)
Eugenol (5µg/mL)
L Menthol (5µg/mL)
Thymol (5µg/mL)
Extract
Control
Extract (+LPS)
Extract (øLPS)

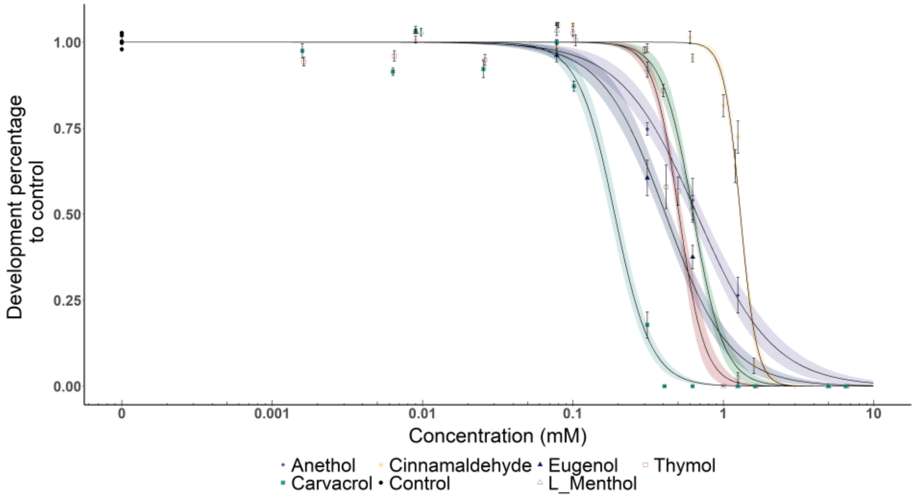
Development percentage
to control
0.00
0.25
0.50
0.75
1.00
0
0.001
0.01
0.1
1
10
Concentration (mM)
Anethol
Cinnamaldehyde
Eugenol
Thymol
Carvacrol
Control
L_Menthol

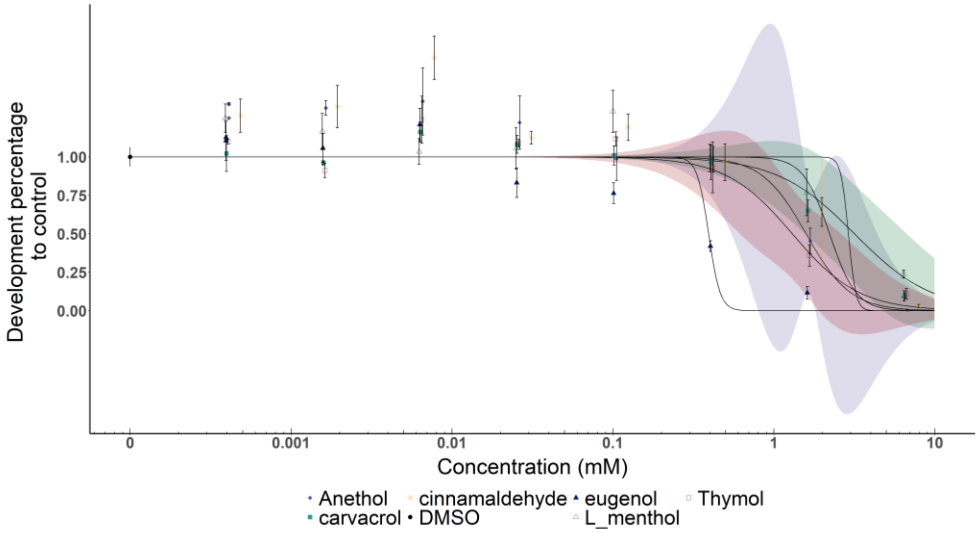
Development percentage
to control
0.00
0.25
0.50
0.75
1.00
0
0.001
0.01
0.1
1
10
Concentration (mM)
Anethol
cinnamaldehyde
eugenol
Thymol
carvacrol
DMSO
L_menthol

Supplementary Figure 1

Effects of terpene compounds on **A**) TNFα production from RAW264.7 cells, **B)** larval development, and **C)** larval migration of equine strongyles.

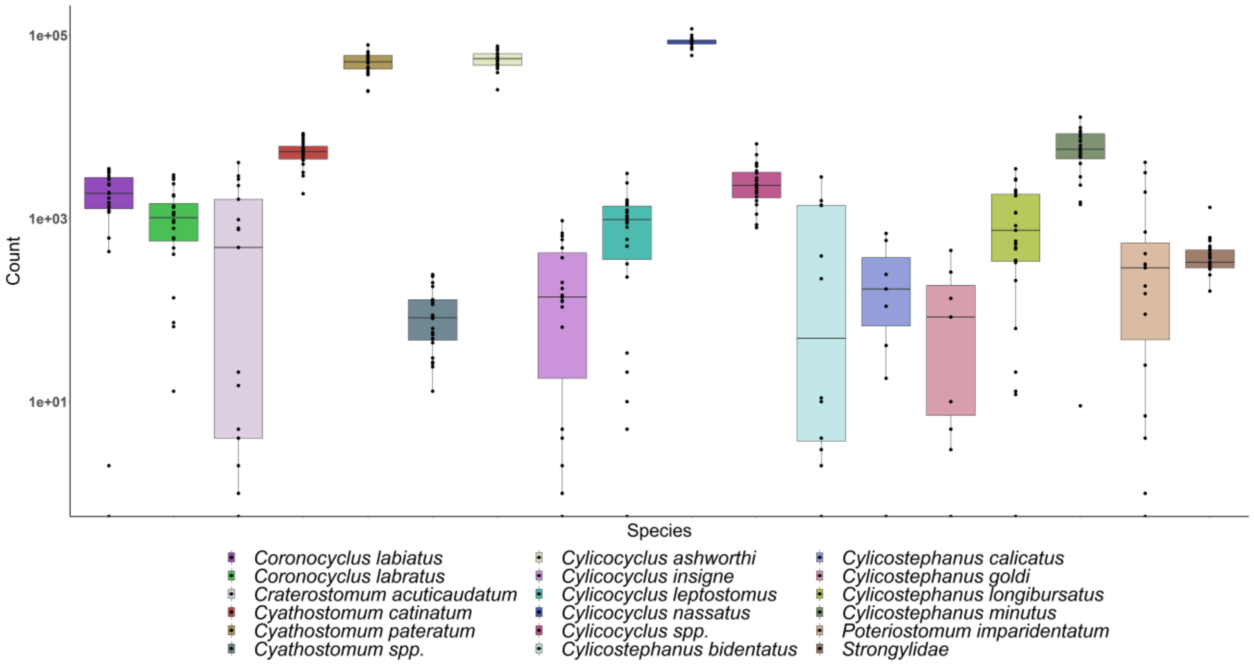

Supplementary Figure 2 - Composition and absolute abundance of species of the isolate studied in the absence of compound (control condition)

Absolute abundance (count) of each species in absence of compound (0 mM). Each point represented the count of individual determine by metabarcoding in each replicate.

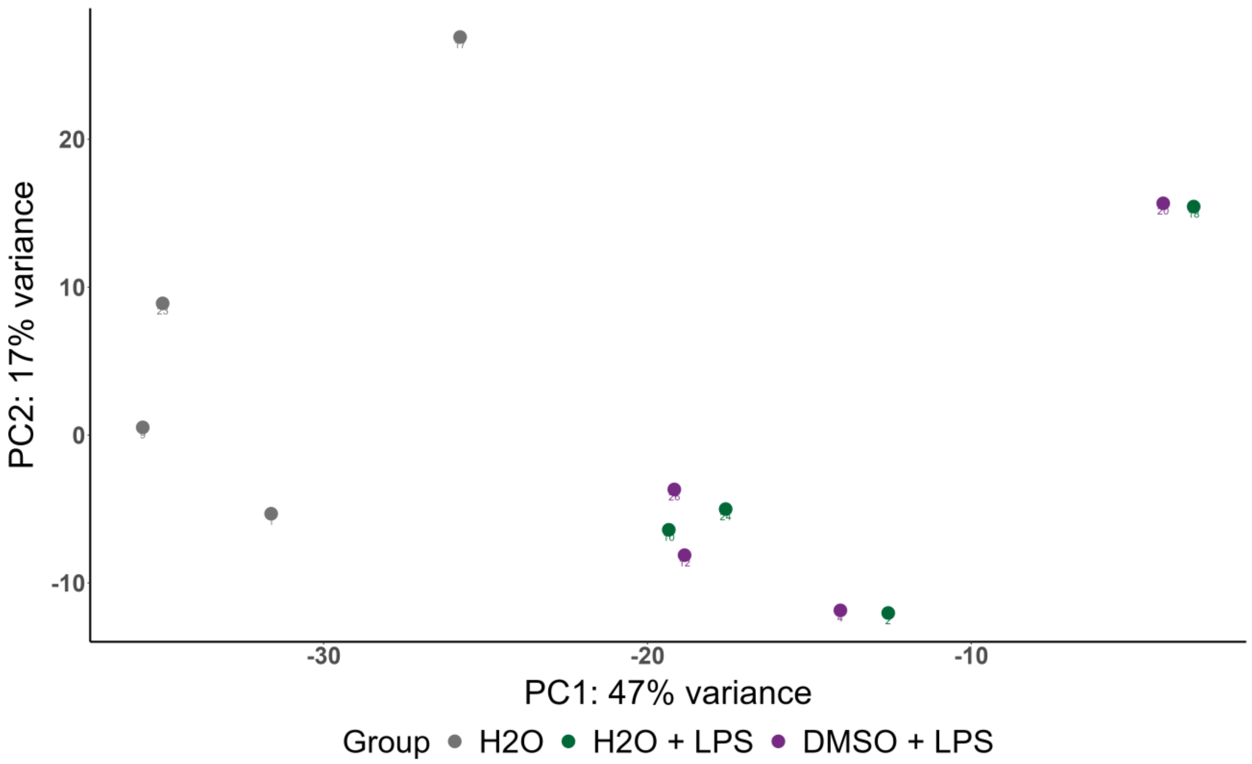

Supplementary Figure 3 – Principal component analysis showing a comparable response to lipopolysaccharide (LPS) treatment in equine monocytes, using either water or DMSO as a solvent.
